# Machine learning models for predicting metabolic syndrome to support clinical decision-making in ART-treated adults living with HIV

**DOI:** 10.1186/s12911-026-03589-9

**Published:** 2026-05-27

**Authors:** Mpanji Siwingwa, Wilbroad Mutale, Sody Mweetwa Munsaka, Violet Kayamba, Douglas C. Heimburger, Scott Hazelhurst, Suilanji Sivile, Aggrey Mweemba, Nyuma Mbewe, Lloyd B. Mulenga, Musalula Sinkala

**Affiliations:** 1 School of Health Sciences, Department of Biomedical Sciences, University of Zambia, Lusaka, Zambia; 2 School of Public Health, Department of Policy, University of Zambia, Lusaka, Zambia; 3 School of Medicine, Department of Internal Medicine, University of Zambia, Lusaka, Zambia; 4 Vanderbilt Institute for Global Health, Vanderbilt University Medical Center, Nashville, TN USA; 5 School of Electrical & Information Engineering, University of the Witwatersrand, Johannesburg, South Africa; 6 Sydney Brenner Institute for Molecular Bioscience, University of the Witwatersrand, Johannesburg, South Africa; 7 Computational Biology Division, Department of Integrative Biomedical Sciences,Faculty of Health Sciences, Institute of Infectious Disease and Molecular Medicine, University of Cape Town, Cape Town, South Africa; 8 Adult Infectious Disease Center, University Teaching Hospital, Lusaka, Zambia; 9 Zambia National Public Health Institute, Lusaka, Zambia

**Keywords:** Metabolic syndrome, HIV, Antiretroviral therapy, Machine learning, Predictive modelling, Calibration, Sub-Saharan Africa

## Abstract

**Background:**

Metabolic syndrome (MetS) is an emerging complication among people living with HIV (PLHIV) receiving long-term antiretroviral therapy (ART), particularly with protease and integrase inhibitor regimens. Early identification of high-risk individuals remains challenging, and predictive tools are limited in African settings. This study evaluated nine machine learning (ML) algorithms for predicting MetS in ART-treated adults.

**Methods:**

We analysed a retrospective cohort of 1,027 PLHIV without baseline MetS, followed for 144 weeks; 854 with complete data were included. Nine ML algorithms, including logistic regression, support vector machines, random forest, and XGBoost, were trained on 70% of the data using stratified repeated 10-fold cross-validation with hyperparameter tuning. Performance was assessed on a 30% test set using discrimination (AUC), calibration, and predictor importance.

**Results:**

Age, sex, viral load, and alcohol use were the strongest predictors of metabolic syndrome in ART-treated individuals. Discrimination was modest across models (AUC 0.49–0.64), with radial SVM and logistic regression performing best (AUC ≈ 0.64). Calibration was generally acceptable (Brier score 0.16–0.19). Sensitivity–specificity trade-offs varied: XGBoost favored sensitivity (85.2%) but had low specificity (34.4%), whereas logistic regression and random forest achieved higher specificity (~ 75%). Overall, complex models offered limited gains over logistic regression.

**Conclusions:**

Although internally validated ML models demonstrated acceptable calibration and modest discrimination, predictive performance remains insufficient for standalone clinical deployment and should be considered supportive rather than definitive for risk stratification in HIV care. Logistic regression and random forest provided the most consistent balance of discrimination and calibration, while complex approaches offered limited gains. Age, sex, viral load, and alcohol use emerged as key predictors. External validation and prospective evaluation are essential to establish generalisability, clinical impact, and feasibility before integration into routine practice.

**Supplementary Information:**

The online version contains supplementary material available at 10.1186/s12911-026-03589-9.

## Background

Cardiometabolic diseases are increasing rapidly worldwide and represent a growing public health challenge, particularly in low- and middle-income regions. Demographic transitions, urbanisation, and shifts in dietary and lifestyle patterns have contributed to a sharp rise in noncommunicable diseases (NCDs). Over the past three decades, cardiovascular-related deaths have increased substantially, rising from just over 12 million in 1990 to approximately 18.6 million in 2019. In sub-Saharan Africa (SSA), cardiovascular diseases are now among the leading contributors to the NCD burden, accounting for a substantial proportion of disability-adjusted life years (DALYs) and premature mortality. As these epidemiological transitions continue, metabolic syndrome (MetS)—a cluster of cardiometabolic risk factors including abdominal obesity, dyslipidaemia, hypertension, and impaired glucose regulation—has emerged as an important driver of cardiovascular morbidity in the region [1, 2].

The prevalence of MetS in SSA has increased steadily over recent decades, reflecting broader shifts in population health and lifestyle patterns. Current estimates suggest that approximately one-quarter of adults in many SSA settings meet diagnostic criteria for MetS, with higher rates reported in urban populations. Southern Africa in particular has been identified as one of the regions with the highest prevalence within the continent [3–5]. These trends are concerning because MetS substantially increases the risk of cardiovascular disease, type 2 diabetes, and other long-term complications [1, 2].

People living with HIV (PLHIV) represent a population that may be particularly vulnerable to metabolic disorders. The widespread scale-up of antiretroviral therapy (ART) has transformed HIV infection from a fatal disease into a manageable chronic condition, enabling PLHIV to live longer and healthier lives. However, this success has been accompanied by new health challenges, including an increasing burden of noncommunicable diseases [6, 7]. Metabolic abnormalities among PLHIV are multifactorial and may result from a combination of aging, lifestyle changes, chronic inflammation associated with HIV infection, and long-term exposure to antiretroviral medications [8]. Recent studies have also reported substantial weight gain following transitions from non-nucleoside reverse transcriptase inhibitors (NNRTIs) to newer antiretroviral regimens, particularly dolutegravir (DTG)-based therapies when combined with tenofovir alafenamide (TAF). These shifts have raised concerns regarding a potential increase in metabolic complications in this population. Weight gain has also been observed in patients receiving protease inhibitor (PI)-based therapies. If not addressed, these metabolic changes may increase the long-term risk of cardiovascular disease, type 2 diabetes, and stroke [7].

Both HIV infection and obesity are associated with changes in adipose immune cell populations and ectopic fat. These changes are associated with metabolic dysregulation. These elements contribute to the increasing burden of metabolic illnesses in the aging HIV population. The risk of metabolic diseases increases with excess adiposity. Furthermore, weight gain in PLHIV confers a greater risk of metabolic disease than in HIV-negative individuals [9, 10].

The successful scale-up of antiretroviral therapy in Zambia has led to a dramatic increase in treatment coverage, from 350,000 people living with HIV (PLHIV) in 2010 to approximately 1.2 million in 2022 [1, 11]. As this population continues to age, the burden of MetS and other NCDs is expected to grow. Recent studies have estimated that approximately one quarter of PLHIV in Zambia meet diagnostic criteria for MetS [1], reflecting a broader trend observed across many African settings [3]. The increasing coexistence of HIV and metabolic disorders presents a significant challenge for healthcare systems, as these conditions require long-term monitoring, prevention strategies, and integrated care approaches [12].

Machine learning (ML) approaches offer promising opportunities for addressing this challenge. Machine learning algorithms are particularly well suited for modelling complex, often nonlinear interactions between clinical variables in HIV patients. Unlike traditional statistical methods, ML can uncover subtle patterns within large, multifactorial datasets to generate robust predictions of future treatment trajectories and complications. In the context of metabolic syndrome, these approaches show promise for stratifying patient risk and anticipating individual care needs before overt symptoms emerge. This capability is critical for advancing differentiated care models, as more precise predictions allow clinicians to detect conditions earlier, target interventions more efficiently, optimise resource allocation, and ultimately improve long-term outcomes by initiating therapy on the basis of a comprehensive assessment of individual risk and potential benefit [12–14].

Therefore, to mitigate MetS, it is essential to identify high-risk individuals who can benefit from tailored preventive and therapeutic measures via MetS prediction models. The aim of this study was to develop and internally validate supervised machine learning models to predict MetS among PLHIV receiving dolutegravir-based antiretroviral therapy in a Zambian cohort.

## Methods

### Study design and setting

This retrospective longitudinal cohort study utilised anonymised clinical data from people living with HIV (PLHIV) receiving care at the University Teaching Hospital in Lusaka, Zambia. Participants were followed for up to 144 weeks to assess the development of metabolic syndrome (MetS) and to develop predictive models using supervised machine learning approaches. The study adhered to the Transparent Reporting of a multivariable prediction model for Individual Prognosis or Diagnosis (TRIPOD) guidelines [15, 16].

### Study population

The study included PLHIV without MetS at baseline who were enrolled in longitudinal follow-up. Only participants with adequate follow-up data over 144 weeks were included. Individuals with MetS at baseline were excluded to ensure that analyses focused on incident MetS during follow-up.

### Outcome definition: metabolic syndrome

MetS was defined using established clinical criteria based on the presence of at least three of the following components: abdominal obesity (waist circumference ≥ 102 cm in men or ≥ 88 cm in women), elevated fasting glucose (≥ 5.6 mmol/L or a diagnosis of diabetes mellitus), reduced high-density lipoprotein cholesterol (< 1.03 mmol/L in men or < 1.29 mmol/L in women), elevated triglycerides (≥ 1.7 mmol/L), and elevated blood pressure (systolic ≥ 130 mmHg or diastolic ≥ 85 mmHg) [17, 18]. A binary outcome variable (incident MetS: yes/no) was constructed. Variables directly used in defining MetS were excluded from the predictor set to prevent target leakage.

### Predictor variables

Predictors included routinely collected demographic, clinical, and behavioural variables: age, sex, antiretroviral therapy (ART) regimen, diabetes status, tobacco use, alcohol consumption, educational level, and viral load. Only variables not included in the MetS definition were considered for model development.

### Data preprocessing

Data preprocessing was conducted in R (version 4.5.1). Viral load was log₁₀-transformed to reduce skewness. Categorical variables were standardised and encoded appropriately, with educational level treated as an ordered factor. Analyses were restricted to follow-up measurements to support longitudinal prediction. Missingness was minimal (0.8%) across predictors and appeared to be approximately random, with no systematic association to outcome or key covariates. Given this very low proportion, complete-case analysis was adopted to maintain methodological simplicity and transparency and avoid introducing additional assumptions. Multiple imputation methods (e.g., MICE) were considered but not implemented, as the negligible level of missingness was unlikely to materially affect estimates or model performance.

### Predictor selection and multicollinearity assessment

A structured approach was used to identify relevant predictors. Random forest–based variable importance was used for initial screening, followed by Boruta feature selection to confirm predictor relevance. Model-agnostic interpretability was assessed using Shapley Additive Explanations (SHAP). Multicollinearity was evaluated using variance inflation factors (VIFs), with all retained predictors showing VIF < 5 (Table 2). Hip circumference was excluded from the primary analysis due to its strong correlation with waist circumference, a diagnostic component of MetS. Detailed results of the Boruta feature selection and random forest variable importance analyses are provided in (*Additional file 2: *Figures S1–S2) including variable ranking, importance scores, and confirmation of selected predictors across methods.

### Model development

**Fig. 1:**
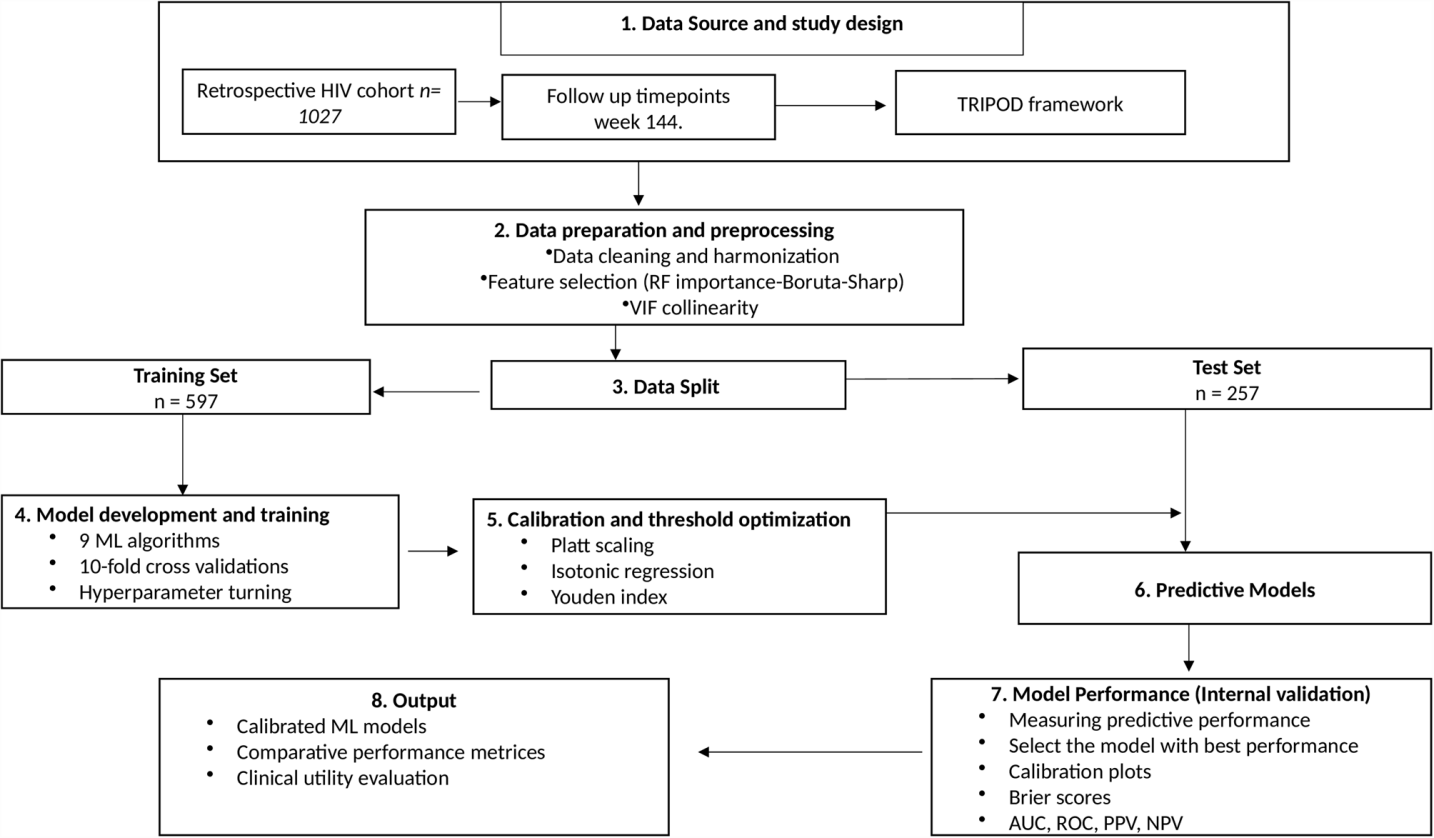
Schematic summarising the development and internal validation processes for predicting metabolic syndrome. The pipeline included data preprocessing, predictor selection, stratified training and test splitting, model training via nine algorithms with 10-fold cross-validation, post-hoc calibration, and comparative performance evaluation to select the optimal model for clinical use

Nine supervised machine learning algorithms were evaluated, including logistic regression, random forest, gradient boosting (XGBoost and LightGBM), support vector machines (linear and radial), decision tree, k-nearest neighbours, and naïve Bayes. The dataset was randomly split into training (70%) and test (30%) sets using stratified sampling to preserve outcome prevalence. Model training was performed on the training set using repeated stratified 10-fold cross-validation with hyperparameter tuning. Final models were re-trained on the full training data and evaluated on the independent test set. Baseline characteristics of participants in the training and test sets are presented in Table 1. Figure 1 summarises the overall machine learning framework, including data preparation, predictor selection, model training, calibration, and performance evaluation.

### Model evaluation and validation

Model performance was assessed on the test set using the outcome distribution. Discrimination was evaluated using the area under the receiver operating characteristic curve (AUC-ROC) and the precision–recall curve (AUC-PR), alongside sensitivity, specificity, and Matthews correlation coefficient. Calibration was assessed using calibration curves and the Brier score. Where miscalibration was observed, post-hoc calibration was performed using Platt scaling and isotonic regression. Classification thresholds were optimised using the Youden index. Clinical utility was evaluated using decision curve analysis across threshold probabilities ranging from 0.05 to 0.30. Confidence intervals for performance metrics were estimated using non-parametric bootstrap resampling (1,000 iterations).

### Sensitivity analysis

A sensitivity analysis was conducted to assess the impact of including hip circumference in model development. Models with and without hip circumference were trained using identical procedures and compared using AUC and Matthews correlation coefficient to evaluate its incremental predictive value. Detailed results of the sensitivity analysis are provided in (*Additional file 1: *Table S1) for model performance including hip circumference, and (*Additional file 1: *Tables S2–S3) for correlation and partial correlation analyses.

### Ethical considerations

This study was approved by the University of Zambia Biomedical Research Ethics Committee (UNZABREC) (Ref: 4887–2024) and the National Health Research Authority (NHRA) (Ref: NHRA1124/15/04/2024). Written permission for secondary data use was obtained from the principal investigator of the VISEND study. All methods were conducted in accordance with relevant ethical guidelines and regulations.

### Statistical software

All analyses were conducted in R (version 4.5.1). A fixed random seed was applied to all stochastic procedures to ensure reproducibility.

## Results

### Participant characteristics

A total of 1,027 participants free of metabolic syndrome (MetS) at baseline were enrolled and followed for 144 weeks. The mean age was 44.1 ± 10.2 years, with a median of 44 years (IQR 38–51). Participants were distributed across four antiretroviral therapy (ART) regimens: AZT+3TC+ATVr (*n* = 167), AZT+3TC+LPVr (*n* = 146), TAFED (*n* = 350), and TLD (*n* = 353). Dolutegravir-based regimens included a higher proportion of individuals aged ≥ 40 years (73–74%) compared with protease inhibitor (PI) regimens (64%). Sex distribution was similar across groups (female: 59–63%). Participants on dolutegravir-based regimens had generally higher CD4 counts and better viral suppression than those on PI regimens. Alcohol use varied across regimens, ranging from 19% to 30%, while diabetes, tobacco use, and education level were comparable across groups.

During follow-up, 182 participants (21.3%) developed MetS. Retention remained high, with ≥ 75% completing follow-up to week 144. Most losses were due to missed visits, and 20 deaths were recorded. The highest number of MetS events occurred in the TAFED, TLD, and PI groups.

#### Baseline characteristics by MetS status

Table 1 summarises baseline characteristics stratified by MetS status in the training and test datasets. In the training set, participants with MetS were more likely to be aged ≥ 40 years (90% vs. 68%, *p* < 0.001). A similar but non-significant trend was observed in the test set (76% vs. 66%, *p* = 0.205). Alcohol use differed significantly in the test set only (15% vs. 30%, *p* = 0.036). No significant differences were observed for sex or viral load suppression. Overall, age showed the most consistent association with MetS across datasets.

**Table 1:** Baseline characteristics by mets status — training and test set

Characteristics	Training Set			Test Set		
	Without MetS	With MetS	*p*-value^2^	Without MetS	With MetS	*p*-value^2^
**MetS**,** n (%)**	471 (78.6%)	128 (21.4%)	1	201 (78.8%)	54 (21.2%)	1
**Age ≥ 40 years**	319 (68%)	115 (90%)	< 0.001	132 (66%)	41 (76%)	0.205
**Viral Load ≥ 1000 cp/mL**	23 (4.9%)	2 (1.6%)	0.133	14 (7.0%)	2 (3.7%)	0.535
**Sex (female)**	298 (63%)	89 (70%)	0.211	123 (61%)	32 (59%)	0.875
**Alcohol Use**	110 (24%)	21 (17%)	0.093	60 (30%)	8 (15%)	0.036

Values are presented as ^1^n (%); Mean (SD), ^2^Pearson’s Chi-squared test;, Fisher’s exact test, and Welch’s two-sample t-test were used as appropriate

Table 1. Baseline characteristics of participants stratified by MetS status in the training and test datasets at 144 weeks. Values are presented as n (%) or mean (SD). Categorical variables were compared using Pearson’s Chi-squared test or Fisher’s exact test (when expected counts < 5), and continuous variables were compared using Welch’s two-sample t-test.

#### Multicollinearity assessment

Variance inflation factors (VIFs) indicated no evidence of multicollinearity among predictors (Table 2). All variables had VIF values below 1.1, well under the conventional threshold of concern (VIF < 5). Sex (1.08) and alcohol use (1.07) showed the highest values, while age and viral load were approximately 1.03. These findings confirm that predictors contributed independently to the models.

**Table 2:** Variance Inflation Factors (VIF)

Predictor Variable	Variance Inflation Factor
Age (years)	1.03
Sex (male/female)	1.08
Alcohol consumption (Yes/No)	1.07
Log Viral load	1.03

Table 2 presents the variance inflation factor (VIF) values used to assess multicollinearity among the predictors in the final regression model. All variables demonstrated VIFs well below the conventional threshold of 5, indicating no evidence of problematic collinearity.

### Model performance

#### Model calibration and probability performance

**Fig. 2:**
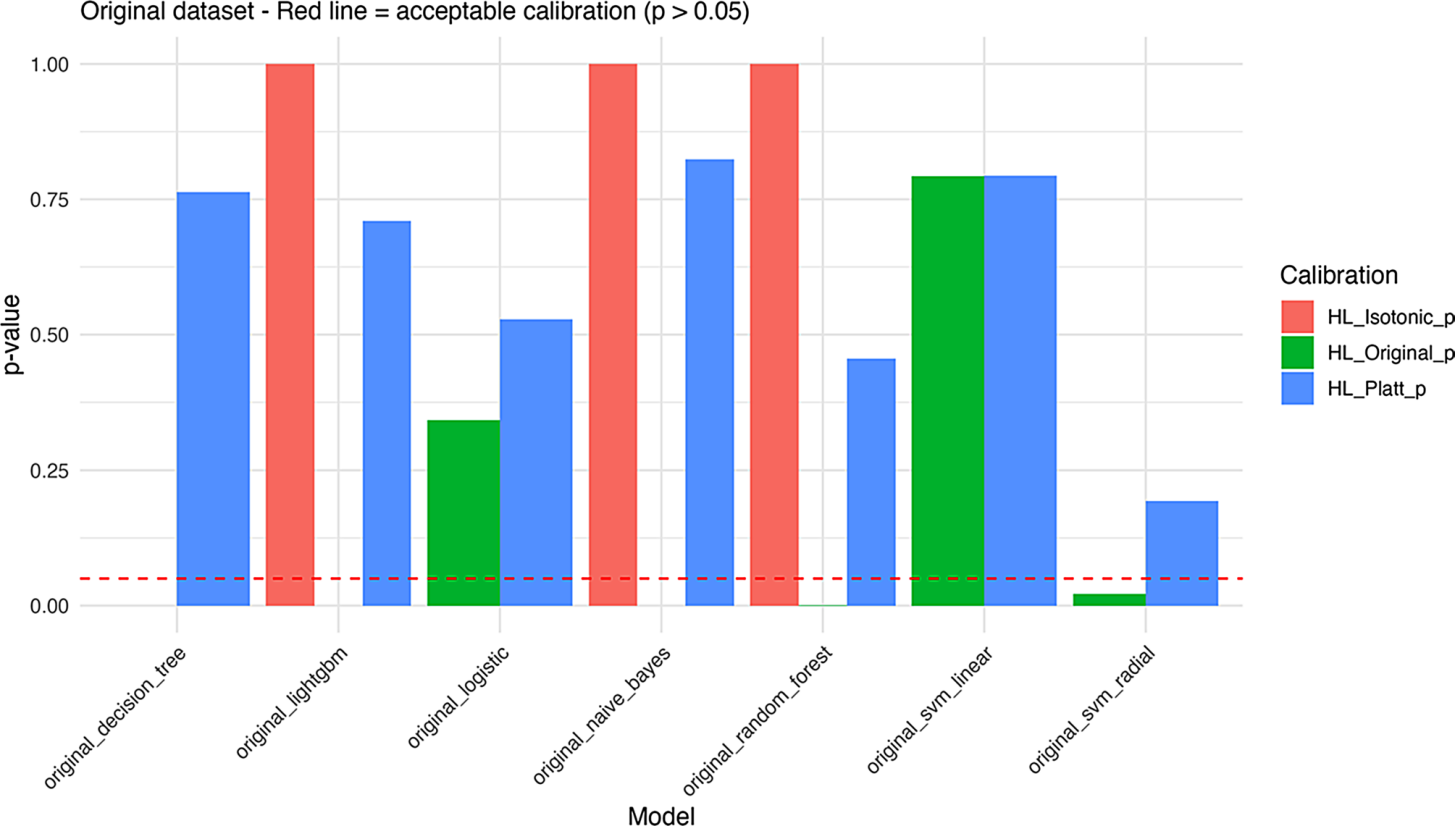
Hosmer–Lemeshow (HL) calibration p-values for machine learning models before and after post-hoc calibration. The dashed red line marks the threshold for acceptable calibration (*p* > 0.05). *Footnote*: HL, Hosmer–Lemeshow; p, p-value

Calibration was assessed using Hosmer–Lemeshow tests, Brier scores, and calibration curves (Fig. 2). Logistic regression and SVM models showed good baseline calibration (HL *p-value > 0.05*). In contrast, tree-based models and naïve Bayes demonstrated significant miscalibration prior to adjustment (HL *p-value < 0.001*). Platt scaling improved calibration across most poorly calibrated models, while isotonic regression was less stable and occasionally failed to converge. K-nearest neighbours was assessed using Brier scores and calibration plots due to discrete probability outputs. Brier scores ranged from 0.16 (best-performing models) to 0.30 (naïve Bayes), indicating moderate overall calibration performance. Logistic regression showed the most stable probability estimates.

#### Discrimination performance (ROC and PR analysis)

**Fig. 3:**
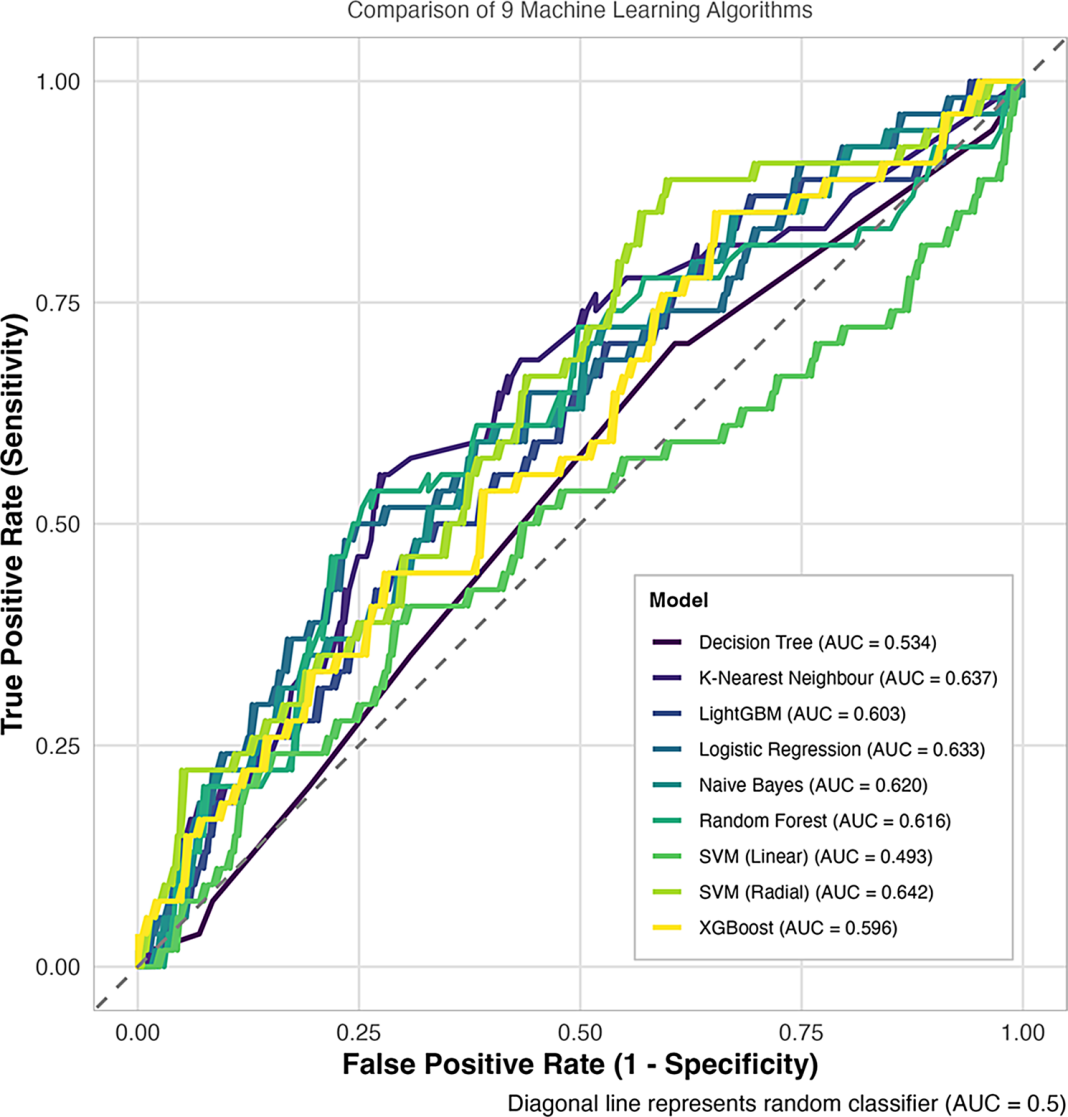
ROC curves comparing classifier performance on the dataset. Curves closer to the top left indicate stronger discrimination. Footnote: ROC, receiver operating characteristic; AUC, area under the curve. The diagonal dashed line represents a random classifier (AUC = 0.5)

ROC analysis demonstrated modest discrimination across all models (Fig. 3). SVM radial achieved the highest AUC (0.642; 95% CI: 0.561–0.723), followed by k-nearest neighbours (0.637; 95% CI: 0.551–0.722) and logistic regression (0.633; 95% CI: 0.548–0.718). Random forest (0.616) and naïve Bayes (0.620) showed intermediate performance. Decision tree (0.534) and SVM linear (0.493) performed poorly. XGBoost achieved an AUC of 0.596 (95% CI: 0.511–0.681), indicating limited discrimination relative to other ensemble methods. Precision–recall AUC (AUC-PR) values ranged from 0.22 to 0.32, reflecting reduced model performance under class imbalance.

#### Internal validation and classification performance

Internal validation results are summarised in Table 3. Logistic regression, k-nearest neighbours, and random forest achieved the highest discrimination, while decision tree and SVM linear performed least well. Sensitivity and specificity varied substantially across models. XGBoost achieved the highest sensitivity (85.2%) but low specificity (34.4%). In contrast, logistic regression (specificity 76.6%) and random forest (75.6%) favoured specificity. K-nearest neighbours showed the most balanced trade-off. Positive predictive values were consistently high (> 80%), while negative predictive values were low (23–35%), indicating limited ability to rule out MetS. Random forest achieved the highest Youden index (0.256), followed by logistic regression (0.229). Optimal thresholds varied widely (0.08–0.62), reflecting differences in model calibration and class separation.

**Table 3:** Performance of machine learning models (Primary analysis excluding hip circumference)

Model Type	AUC (95% CI)	AUC_PR (%)	Brier Scores	Sensitivity (%)	Specificity (%)	PPV (%)	NPV (%)	Accu (%)	Youden Index	Optimal Threshold
Decision tree	53.4 (45.1, 61.8)	22.2	0.19 (0.16, 0.23)	70.4	37.8	82.6	23.3	44.7	0.082	0.08
K-NN	63.7 (55.1, 72.2)	29.9	0.18 (0.14, 0.21)	55.6	72.6	85.9	35.3	69	0.282	0.21
LightGBM	60.3 (51.9, 68.7)	26.6	0.19 (0.15, 0.22)	70.4	46.8	85.5	26.2	51.8	0.171	0.10
Logistic Regression	63.3 (54.8, 71.8)	32.1	0.16 (0.13, 0.19)	46.3	76.6	84.2	34.7	70.2	0.229	0.25
Naïve Bayes	62 (53.6, 70.4)	28.2	0.3 (0.26, 0.34)	59.3	62.2	85.0	29.6	61.6	0.214	0.62
XGBoost	59.6 (51.1, 68.1)	30.7	0.18 (0.14, 0.21)	85.2	34.4	89.7	26	45.5	0.2	0.09
Random Forest	61.6 (52.6, 70.5)	28.5	0.18 (0.14, 0.2)	50.0	75.6	84.9	35.5	70.2	0.256	0.25
SVM Linear	49.3 (39.8, 58.9)	22.0	0.17 (0.14, 0.2)	24.1	83.6	80.4	28.3	71	0.077	0.22
SVM Radial	64.2 (56.1, 72.3)	31.8	0.16 (0.14, 0.19)	72.2	46.8	86.2	26.7	52.2	0.190	0.21

#### Decision curve analysis

**Fig. 4:**
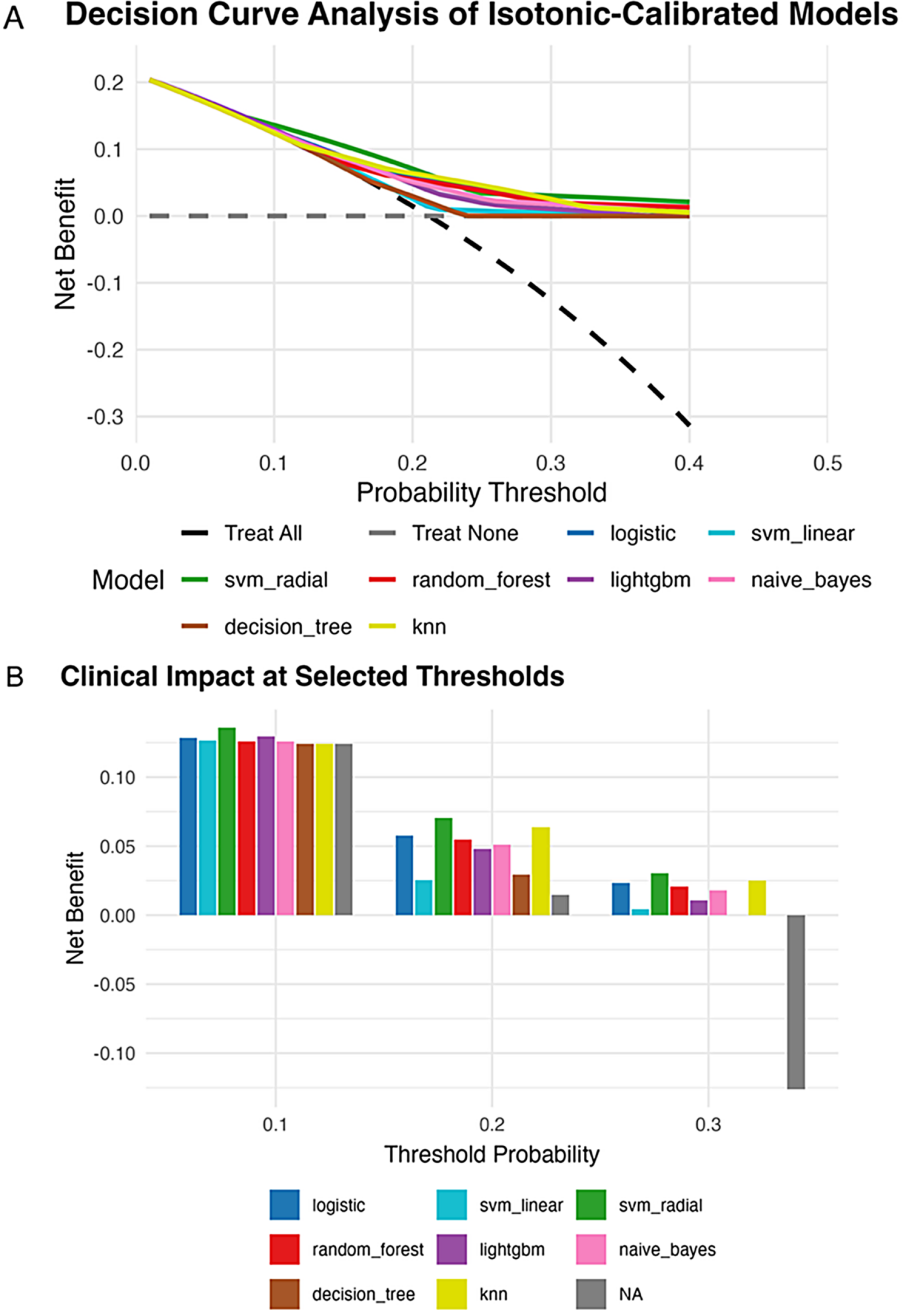
Decision curve analysis. panel a shows net benefit curves for eight isotonic-calibrated models across thresholds (0–1). Panel B illustrates model performance for predicting metabolic syndrome. “Treat None” (black dashed) and “Treat All” (red solid) serve as reference strategies

Decision curve analysis (Fig. 4) demonstrated that several models provided clinical net benefit across probability thresholds of 0.05–0.30. At a threshold of 0.10, random forest achieved the highest net benefit (0.163), closely followed by logistic regression (0.161) and SVM linear (0.159). Naïve Bayes and SVM radial showed similar intermediate performance. XGBoost and LightGBM demonstrated moderate net benefit, while k-nearest neighbours showed consistently low clinical utility. Overall, random forest, logistic regression, and SVM-based models provided the most consistent net benefit across clinically relevant thresholds.

### Feature importance and interpretation

#### Predictor importance and secondary analyses

**Fig. 5:**
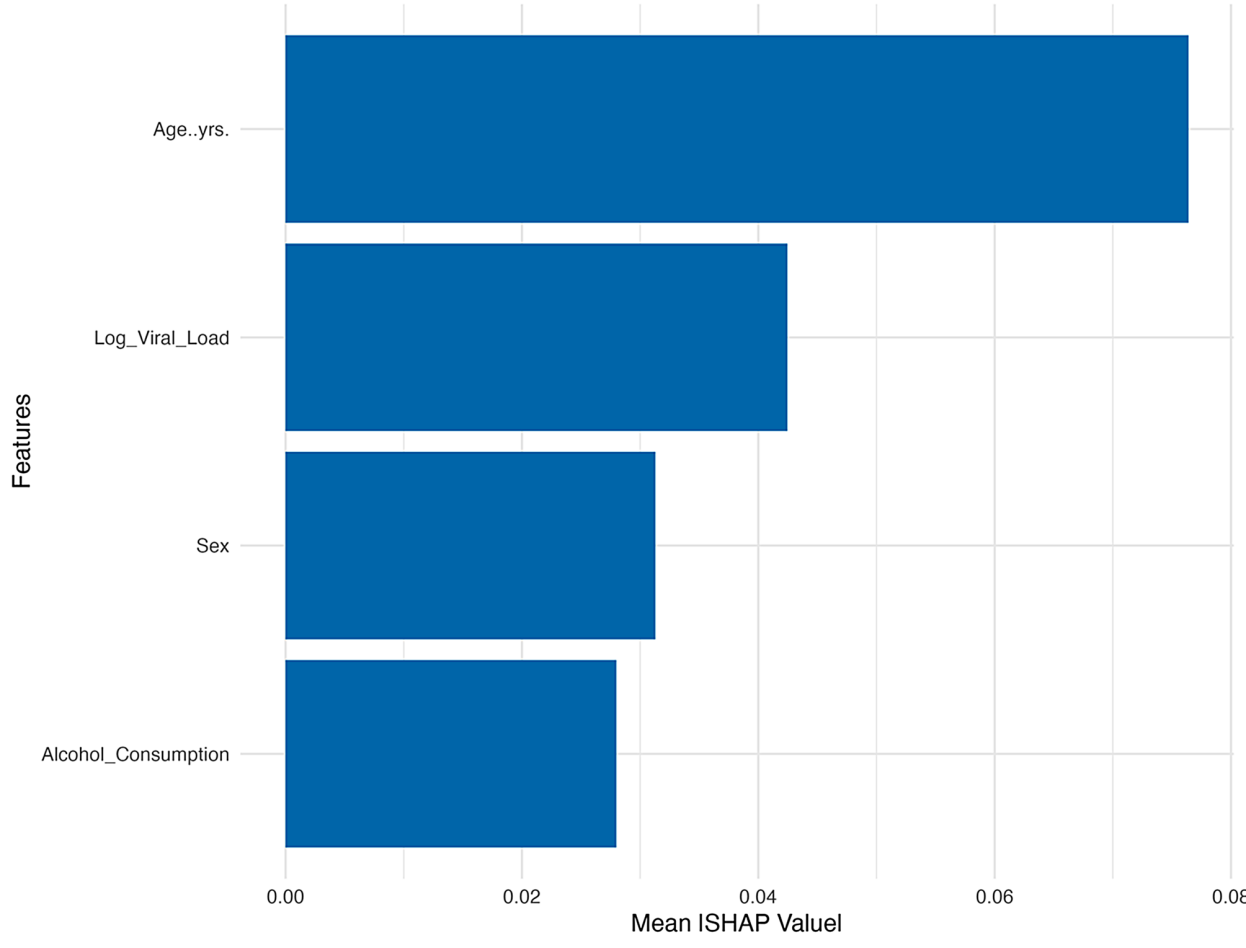
Top-ranked predictors by mean absolute SHAP values from the final model. SHAP analysis quantified the relative contribution of each predictor, providing model-agnostic interpretability and highlighting the variables with the greatest influence on classification outcomes

SHAP analysis identified age as the strongest predictor of MetS, followed by viral load, sex, and alcohol use (Fig. 5). These variables consistently contributed most to model predictions across algorithms. Pearson correlation analysis showed a moderate positive correlation between waist and hip circumference (*r* = 0.695, *p* < 0.001). After adjustment for waist circumference, hip circumference was no longer independently associated with MetS (partial *r* = − 0.02, *p* = 0.57).

Using both generalized additive models (GAM) and logistic regression with an interaction term, CD4 count showed a modest linear association with MetS (*p* = 0.018), while viral load demonstrated an independent but weaker effect (*p* = 0.039). No significant interaction was observed between CD4 count and viral load (*p* = 0.24). VIF values confirmed low collinearity (all < 1.5). The relationship between CD4 count and metabolic syndrome is illustrated in (*Additional file 2: *Figure S3).

#### Sensitivity analysis: effect of hip circumference

**Fig. 6:**
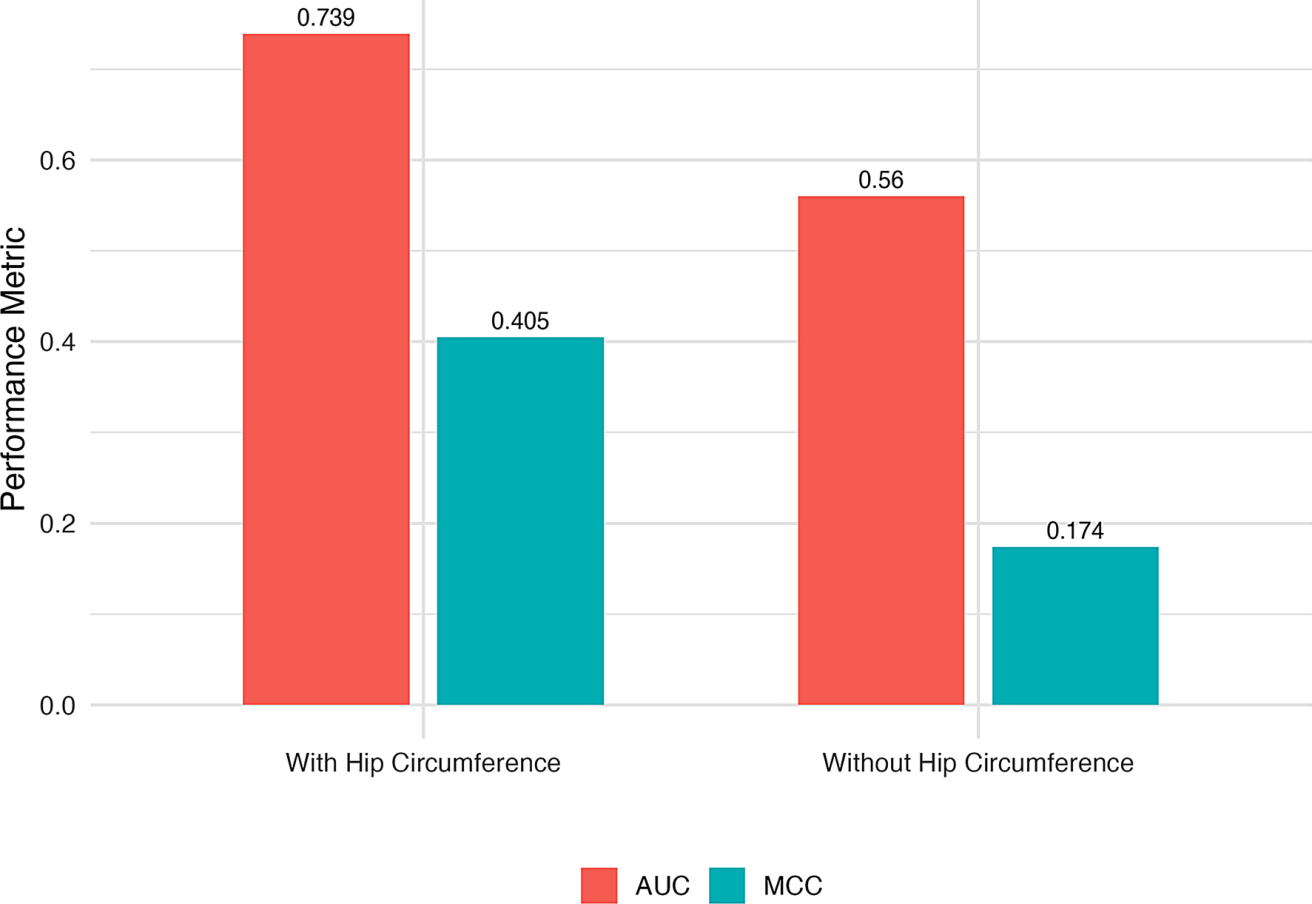
Sensitivity analysis of hip circumference inclusion on model performance. Models trained with hip circumference demonstrated markedly higher discrimination and classification consistency compared with models excluding this variable

To evaluate the influence of hip circumference, models were re-trained with and without this variable. Inclusion of hip circumference substantially improved apparent discrimination, with AUC increasing from 0.56 to 0.74 and MCC from 0.174 to 0.405 (Fig. 6). However, hip and waist circumference were strongly correlated (Pearson *r* = 0.695; 95% CI: 0.659–0.729), and the association with metabolic syndrome was no longer significant after adjusting for waist circumference (partial *r* = − 0.02; *p* = 0.57). These findings indicate that hip circumference enhances model performance largely through shared variance with waist circumference, a diagnostic component of metabolic syndrome. Accordingly, the model excluding hip circumference is reported as the primary analysis, while the model including it represents an upper-bound estimate.

## Discussion

### Key findings

This study developed and internally validated a machine learning framework for predicting metabolic syndrome (MetS) among PLHIV receiving antiretroviral therapy. Overall discriminatory performance was modest, with AUC values ranging from 0.49 to 0.64. SVM (radial) (AUC 0.64), k-nearest neighbours (AUC 0.64), and logistic regression (AUC 0.63) achieved the highest discrimination, followed by random forest (AUC 0.62), while decision tree and SVM (linear) performed least well. Calibration was generally acceptable for most models (Brier scores 0.16–0.19), although Naïve Bayes demonstrated poorer calibration (0.30). Performance trade-offs varied across models. Logistic regression and random forest achieved higher specificity (76.6% and 75.6%, respectively) with moderate sensitivity, whereas XGBoost prioritised sensitivity (85.2%) at the expense of specificity (34.4%). K-nearest neighbours provided a more balanced sensitivity–specificity profile and achieved the highest Youden index (0.28), indicating the best overall diagnostic trade-off. Positive predictive values were consistently high (> 80%) across models, while negative predictive values remained low. These findings suggest that simpler models, particularly logistic regression, can perform comparably to more complex machine learning approaches in this setting. Overall, model selection should be guided by the intended clinical use case and the relative importance of sensitivity versus specificity, rather than discrimination alone [19–22].

### Predictor importance

Consensus predictor importance analysis, based on SHAP values and permutation-based importance across models, identified age, sex, viral load, and alcohol use as the most consistent predictors of MetS. Age emerged as the strongest contributor, reinforcing its central role in metabolic risk among people living with HIV. These findings highlight the multifactorial nature of MetS, where demographic, behavioural, and virological factors jointly influence disease risk. The dominance of age and adiposity-related variables is consistent with established evidence on metabolic dysfunction in both general and HIV populations [23, 24]. Together, these results reinforce the importance of integrating clinical, behavioural, and treatment-related factors in risk prediction models.

### Hip circumference sensitivity analysis

A key methodological consideration relates to the inclusion of hip circumference as a predictor. Although not part of the formal diagnostic criteria for MetS, hip circumference showed a strong correlation with waist circumference (Pearson *r* = 0.695; *p* < 0.001), a core diagnostic component of the outcome. After adjustment for waist circumference, hip circumference was not significantly associated with MetS (partial *r* = -0.02; *p* = 0.57), suggesting limited independent contribution beyond central adiposity. To evaluate its impact on predictive performance, models were developed with and without hip circumference. Inclusion of this variable substantially improved discrimination (AUC 0.74; MCC 0.40), whereas exclusion resulted in reduced performance (AUC 0.56; MCC 0.17). This difference likely reflects both shared variance with waist circumference and partial proxy effects for central adiposity. Accordingly, hip circumference appears to enhance predictive performance but may partly reflect overlap with a diagnostic component of MetS. Therefore, the model excluding hip circumference is presented as the primary and more conservative estimate of generalisable discrimination, while the full model represents an upper-bound performance scenario. Detailed sensitivity analyses are presented in *(Additional file 1: *Table S1) (model performance with hip circumference) and (*Additional file 1: *Tables S2–S3) (correlation analyses).

### Logistic regression versus machine learning models

Although logistic regression demonstrated the highest discrimination among the primary models, its performance was broadly comparable to that of more complex machine learning approaches, including random forest and k-nearest neighbours. This aligns with previous studies showing that logistic regression often performs competitively in clinical prediction tasks, particularly when sample sizes are moderate and predictor structures are well defined [25, 26].

In such settings, machine learning approaches may not yield substantial performance gains unless large datasets and extensive tuning are available. These findings are consistent with established methodological guidance by Steyerberg, who emphasises that logistic regression remains a robust and interpretable method in clinical prediction modelling [27]. Overall, these results suggest that differences between models are relatively small and should be interpreted in the context of clinical applicability. Model selection should therefore balance interpretability, calibration, and intended clinical use rather than relying solely on discrimination metrics.

### Comparison with external literature

Anthropometric measures have consistently been identified as strong predictors of metabolic syndrome in previous machine learning studies. Variables such as body mass index, waist circumference, and triglyceride-based indices have shown strong predictive value across different populations [28]. Similarly, measures of body fat distribution, including waist-related indices and body roundness measures, have been shown to improve classification of metabolic risk [29, 30].

In addition, we observed that CD4 count showed a modest association with MetS, independent of viral load. Higher CD4 counts were more frequently observed among individuals with MetS, consistent with previous reports suggesting that immune recovery in ART-treated populations may be associated with increased metabolic risk, potentially due to prolonged treatment exposure and immune reconstitution effects [31]. These mechanisms may involve persistent inflammation and adipose tissue dysfunction despite viral suppression [32–34]. These findings underscore the importance of continued cardiometabolic monitoring in patients who demonstrate successful immune recovery during ART. Alcohol use appeared less frequent among individuals with MetS in descriptive analyses; however, this likely reflects measurement limitations, including self-report bias and binary categorisation, rather than a protective association [35].

### Implications

From a clinical informatics perspective, these findings suggest that machine learning models could support earlier identification of individuals at risk of metabolic syndrome prior to clinical diagnosis. However, as the models were internally validated within a single cohort, performance may be optimistic and requires confirmation in external populations. Validation in independent cohorts, particularly within sub-Saharan Africa, will be essential to confirm generalisability [15, 16, 36].

Because predictors were derived from routinely collected clinical and anthropometric data, implementation may be feasible in resource-limited settings without additional laboratory testing. This enhances scalability within routine HIV programmes, although formal evaluation of feasibility, cost-effectiveness, and workflow integration will be required before clinical deployment [37, 38].

Feature selection was performed using a three-step framework combining random forest importance, Boruta, and SHAP analysis. While this approach improves robustness, each method has limitations, particularly bias toward certain variable types or sensitivity to model structure. Therefore, predictor rankings should be interpreted in combination with clinical knowledge to ensure alignment with established HIV care practices.

From a clinical translation perspective, the comparable performance of logistic regression offers important advantages for implementation. Unlike more complex machine learning models, logistic regression can be readily translated into a simple risk score or probability-based calculator using routinely collected clinical variables. Such a model could be integrated into electronic medical record (EMR) systems or clinical decision support systems (CDSS) to enable automated risk estimation during routine patient visits. This would allow clinicians to identify individuals at high risk of metabolic syndrome in real time and support early, targeted interventions within existing HIV care workflows. Given its interpretability, transparency, and low computational requirements, logistic regression represents a practical and scalable approach for implementation in resource-limited settings. Taken together, although internally validated models demonstrated acceptable calibration and modest discrimination, predictive performance remains insufficient for standalone clinical deployment and should currently be considered supportive rather than definitive for risk stratification.

### Strengths and limitations

This study has several strengths. It focuses on people living with HIV receiving contemporary antiretroviral therapy, a population at increasing risk of cardiometabolic complications but still underrepresented in predictive modelling studies. Multiple machine learning algorithms were systematically evaluated using discrimination, calibration, and decision curve analysis, providing a comprehensive assessment of predictive performance [39].

Several limitations should be considered. First, external and prospective validation is required before clinical application. Second, important predictors such as diet, physical activity, and genetic variables were not available and may improve performance if included. Third, generalisability may be limited due to differences in population characteristics and treatment regimens across settings. Fourth, calibration was assessed using the Hosmer–Lemeshow test, which has known limitations; therefore, calibration curves and Brier scores were also used for more robust assessment. Finally, lack of suitable external datasets limited external validation.

### Future directions

Future work should focus on external validation of these models in diverse HIV cohorts. Incorporating additional data sources, including genetic, immunological, and lifestyle variables, may improve predictive accuracy. Development of user-friendly clinical decision support tools will be important for translation into practice. Prospective evaluation in routine care settings will be necessary to determine whether these models improve early identification and prevention of cardiometabolic complications in people living with HIV [40].

## Conclusions

This study developed and internally validated ML models to predict MetS among PLHIV receiving long-term ART. Overall, model performance was modest, with logistic regression and random forest providing the most consistent balance between discrimination, calibration, and clinical utility. Notably, simpler models performed comparably to more complex ML approaches, highlighting the importance of model interpretability and reliable risk estimation over incremental gains in discrimination. Key predictors—including age, sex, viral load, and alcohol use—reflect the multifactorial nature of metabolic risk in this population. Although these findings support the potential use of predictive models for risk stratification in HIV care, the observed performance indicates limited standalone clinical utility. External validation in independent cohorts and prospective evaluation in routine care settings are required to establish generalisability, clinical impact, and feasibility prior to implementation.

## Supplementary Information

Below is the link to the electronic supplementary material.

Supplementary Material 1

Supplementary Material 2

## Data Availability

The data supporting the findings of this study are available from the corresponding author upon reasonable request. The data supporting the findings of this study contain sensitive clinical information and are therefore not publicly available. Interested parties may contact Mpanji Siwingwa at [mpanjisiwingwa@gmail.com] or Dr. Musalula Sinkala [smsinks@gmail.com] to request access to the data. All requests will be reviewed to ensure compliance with ethical and data protection guidelines.

## Code availability

The complete analysis code used for data preprocessing, feature engineering, model development, calibration, and evaluation is publicly available in the GitHub repository: https://github.com/MpanjiSiwingwa/Metaboic-syndrome-prediction-using-machine-learning/tree/v1.0.0. The repository has been archived and assigned a permanent DOI through Zenodo to ensure long-term accessibility: 10.5281/zenodo.20145268.

## Abbreviations

AIDS: Acquired immune deficiency syndrome
ART: Antiretroviral therapy
AUC: Area under the curve
AUC-ROC: Area under the receiver operating characteristic curve
AUC-PR: Area Under the Precision–Recall Curve
AZT+3TC+ATVr: Zidovudine + Lamivudine + Atazanavir/ritonavir
AZT+3TC+LPVr: Zidovudine + Lamivudine + Lopinavir/ritonavir
BMI: Body mass index
cART: Combination antiretroviral therapy
CSV: Comma-separated value
CVDs: Cardiovascular diseases
DALYs: Disability-adjusted life years
DCA: Decision curve analysis
DT: Decision tree
DTG: Dolutegravir
HDL: High-density lipoprotein
HIV: Human immunodeficiency virus
INSTI: Integrase Strand Transfer Inhibitor
KNN: k-nearest neighbours
LR: Logistic regression
MetS: Metabolic syndrome
ML: Machine learning
NB: Naïve Bayes
NCDs: Noncommunicable diseases
NNRTIs: Non-nucleoside reverse transcriptase inhibitors
NPV: Negative predictive value
PLHIV: People living with HIV
PPV: Positive predictive value
pROC: Package for ROC analysis
RF: Random forest
SSA: Sub-Saharan Africa
SVM: Support vector machines (linear and radial)
TAFED: Tenofovir alafenamide + Emtricitabine + Dolutegravir
TLD: Tenofovir disoproxil fumarate + Lamivudine + Dolutegravir
VIF: Variance inflation factor
XGBoost: Extreme gradient boosting
