# Supplementary material for: Machine learning models for predicting metabolic syndrome to support clinical decision-making in ART-treated adults living with HIV
Source: BMC Med Inform Decis Mak. 2026 May 27;26:299. doi: 10.1186/s12911-026-03589-9 (PMC13450551; doi:10.1186/s12911-026-03589-9)
Supplement: Supplementary file 1 [file 12911_2026_3589_MOESM1_ESM.pdf]

Supplementary Materials

This supplementary material provides additional analyses supporting the main findings. These include sensitivity analyses evaluating the inclusion of hip circumference in model development, as well as correlation and partial correlation analyses assessing its relationship with waist circumference and metabolic syndrome.

Supplementary Table S1. Performance of machine learning models including hip circumference (sensitivity analysis compared with primary models excluding hip circumference)

Model Type	AUC (%)	Brier Score	Optimal Threshold	Sensitivity (%)	Specificity (%)	PPV (%)	NPV (%)	Accuracy (%)	Youden Index
Random Forest	76.0 (69.4, 82.5)	0.14 (0.11, 0.17)	0.18	57.4	80.1	87.5	43.7	75.3	0.375
XGBoost	72.2 (64.3, 80.1)	0.14 (0.11, 0.17)	0.16	75.9	63.2	90.7	35.7	65.9	0.391
SVM-Radial	74.5 (67.2, 81.9)	0.14 (0.11, 0.17)	0.2	61.1	74.6	87.7	39.3	71.8	0. 311
LightGBM	74.7 (67.2, 82.2)	0.14 (0.11, 0.17)	0.16	64.8	73.6	88.6	39.8	71.8	0. 384
Logistic Regression	76.6 (70.0, 83.2)	0.14 (0.11, 0.16)	0.06	90.7	50.2	95.3	32.9	58.8	0.41
SVM-Linear	73.2 (67.0, 80)	0.14 (0.12, 0.17)	0.21	81.5	57.2	92.0	33.8	62.4	0. 387
Naïve Bayes	72.7 (65.5, 79.9)	0.15 (0.12, 0.17)	0.15	75.9	60.7	90.4	34.2	63.9	0.366
Decision Tree	73 (66, 80.)	0.15 (0.12, 0.18)	0.12	64.8	77.1	89.1	43.2	74.5	0. 419
k-NN	75.8 (68.7, 82.9)	0.14 (0.12, 0.17)	0.21	72.2	65.2	89.7	35.8	66.7	0. 374

Supplementary Table S2

Supplementary Table S2. Pearson correlation between waist and hip circumference

Variable 1	Variable 2	Correlation r	95% CI Lower	95% CI Upper	p-value
Waist circumference	Hip circumference	0.695	0.659	0.729	< 0.001

CI confidence interval. Correlation calculated using Pearson's method.

Waist and hip circumference were strongly correlated, indicating substantial shared variance

Supplementary Table S3

Supplementary Table S3. Partial correlation between hip circumference and metabolic syndrome controlling for waist circumference

Variable 1	Variable 2	Control variable	Partial r	p-value
Hip circumference	Metabolic syndrome (MetS)	Waist circumference	-0.02	0.57

MetS defined according to standard criteria [MetS defined according to NCEP ATP III criteria]. Partial correlation adjusted for waist circumference as indicated.

Variable 1	Variable 2	Control variable	Partial r	p-value

After adjustment for waist circumference, hip circumference was not independently associated with metabolic syndrome.
