# Supplementary material for: Machine learning models for predicting metabolic syndrome to support clinical decision-making in ART-treated adults living with HIV
Source: BMC Med Inform Decis Mak. 2026 May 27;26:299. doi: 10.1186/s12911-026-03589-9 (PMC13450551; doi:10.1186/s12911-026-03589-9)
Supplement: Supplementary file 2 [file 12911_2026_3589_MOESM2_ESM.pdf]

Supplementary Figures

These figures provide additional visualisation of model development, including feature importance and the relationship between key clinical variables and metabolic syndrome

Figure S1 Random Forest

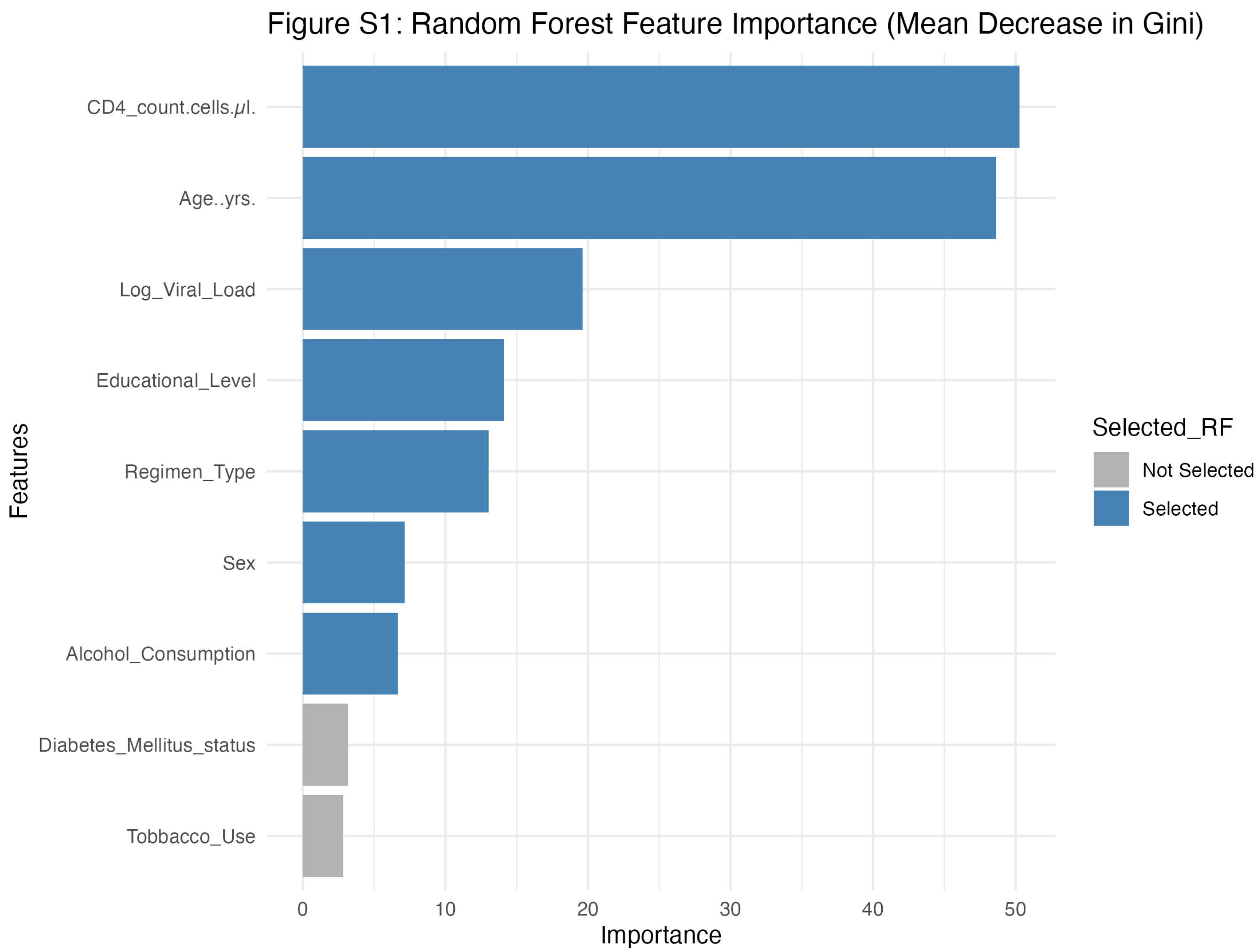

Figure S1 Random forest variable importance for metabolic syndrome prediction.

Variable importance was assessed using mean decrease in Gini impurity. Age and CD4 count were the most influential predictors, followed by viral load and educational level.

Figure S2 Feature (Boruta / Feature selection)

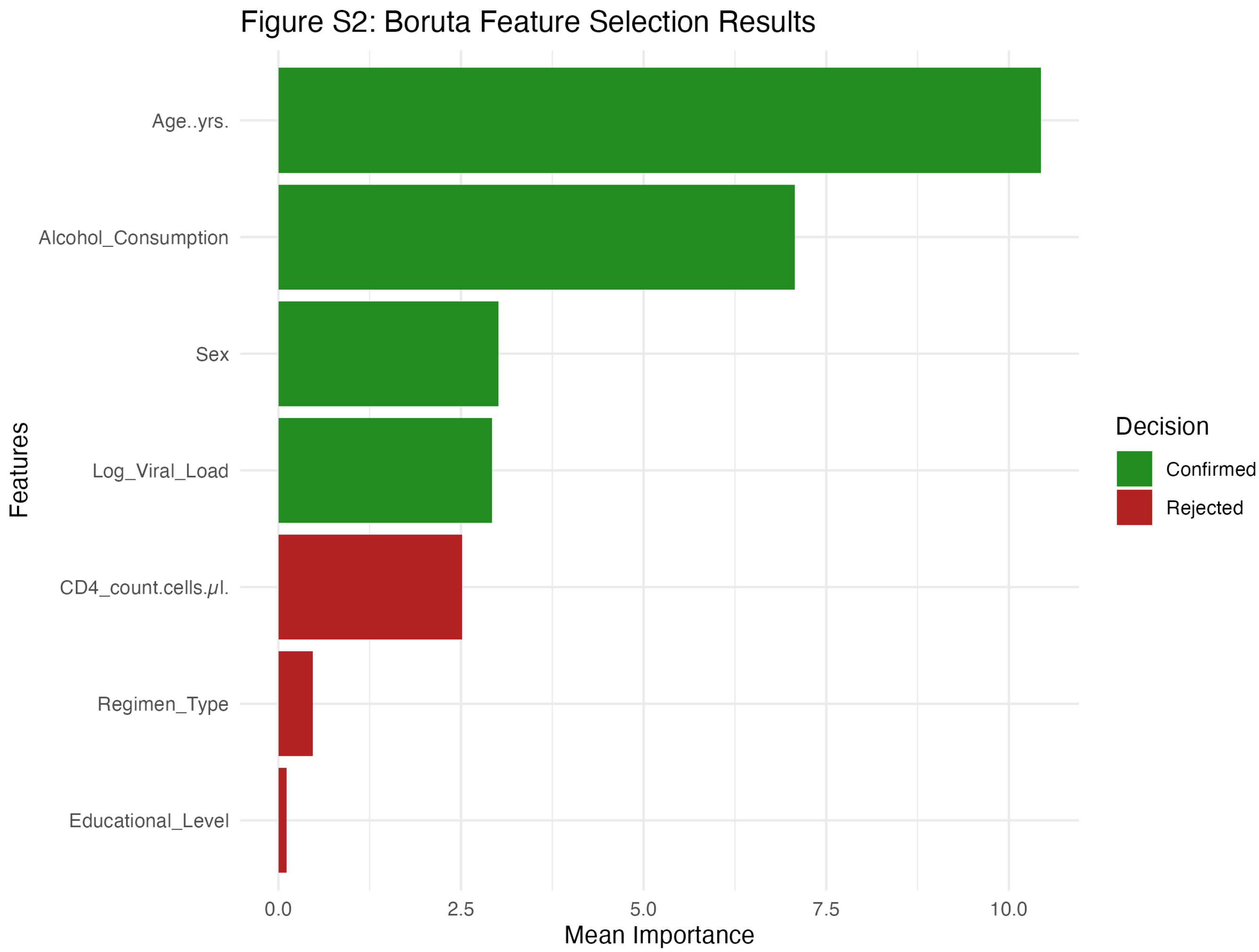

Figure S2 Feature importance of confirmed predictors identified by Boruta.

Variables confirmed as important predictors are shown with their relative importance scores. Age and alcohol consumption were the most influential predictors, followed by sex, log viral load, and CD4 count.

Figure S3: (CD4 relationship)

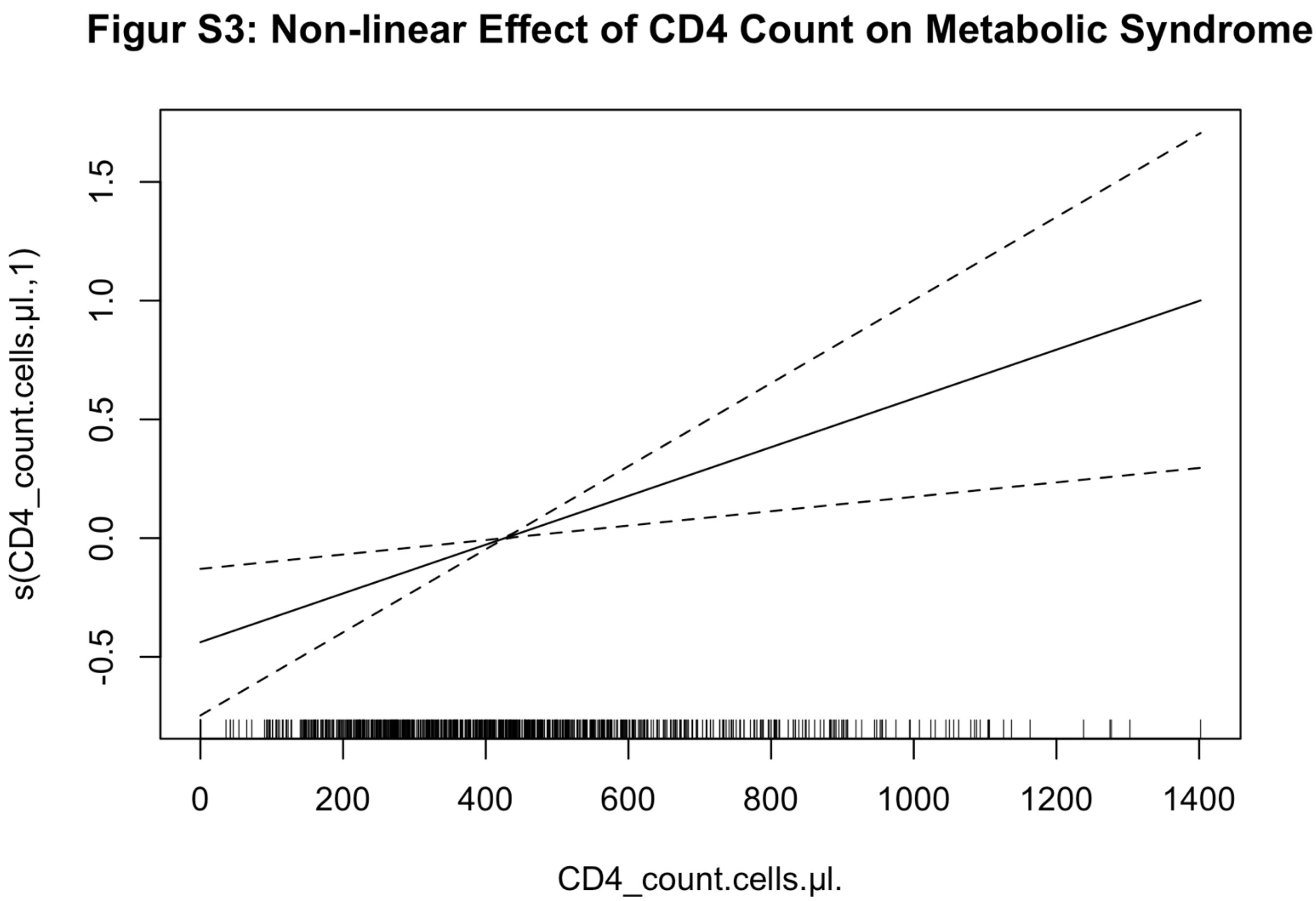

Figure S3. Nonlinear association between CD4 count and metabolic syndrome.

A generalized additive model demonstrated a nonlinear relationship between CD4 count and metabolic syndrome risk. The solid line represents the estimated effect, with dashed lines indicating 95% confidence intervals.
